# Mitochondrial Dysfunction in a High Intraocular Pressure-Induced Retinal Ischemia Minipig Model

**DOI:** 10.3390/biom12101532

**Published:** 2022-10-21

**Authors:** Michael Pasák, Marie Vanišová, Lucie Tichotová, Jana Křížová, Taras Ardan, Yaroslav Nemesh, Jana Čížková, Anastasiia Kolesnikova, Ruslan Nyshchuk, Natasha Josifovska, Lyubomyr Lytvynchuk, Miriam Kolko, Jan Motlík, Goran Petrovski, Hana Hansíková

**Affiliations:** 1 Laboratory for Study of Mitochondrial Disorders, Department of Paediatrics and Inherited Metabolic Disorders, First Faculty of Medicine, Charles University and General University Hospital in Prague, 12801 Prague, Czech Republic; 2 Laboratory of Cell Regeneration and Cell Plasticity, Institute of Animal Physiology and Genetics AS CR, 277 21 Libechov, Czech Republic; 3 Department of Cell Biology, Faculty of Science, Charles University,12808 Prague, Czech Republic; 4 Center for Eye Research, Department of Ophthalmology, Institute of Clinical Medicine, Faculty of Medicine, University of Oslo and Oslo University Hospital, 0450 Oslo, Norway; 5 Department of Ophthalmology, Justus Liebig University, University Hospital Giessen and Marburg GmbH, 35392 Giessen, Germany; 6 Karl Landsteiner Institute for Retinal Research and Imaging, 1030 Vienna, Austria; 7 Eye Translational Research Unit, Department of Drug Design and Pharmacology, Faculty of Health and Medical Sciences, University of Copenhagen, 2100 Copenhagen, Denmark; 8 Department of Ophthalmology, Copenhagen University Hospital, Rigshospitalet, 2600 Glostrup, Denmark; 9 Department of Ophthalmology, University Hospital of Split and University of Split, 21000 Split, Croatia

**Keywords:** minipig, retinal ischemia, mitochondrial dysfunction, coenzyme Q10

## Abstract

**Purpose:** Retinal ischemia (RI) and progressive neuronal death are sight-threatening conditions. Mitochondrial (mt) dysfunction and fusion/fission processes have been suggested to play a role in the pathophysiology of RI. This study focuses on changes in the mt parameters of the neuroretina, retinal pigment epithelium (RPE) and choroid in a porcine high intraocular pressure (IOP)-induced RI minipig model. **Methods:** In one eye, an acute IOP elevation was induced in minipigs and compared to the other control eye. Activity and amount of respiratory chain complexes (RCC) were analyzed by spectrophotometry and Western blot, respectively. The coenzyme Q10 (CoQ10) content was measured using HPLC, and the ultrastructure of the mt was studied via transmission electron microscopy. The expression of selected mt-pathway genes was determined by RT-PCR. **Results:** At a functional level, increased RCC I activity and decreased total CoQ10 content were found in RPE cells. At a protein level, CORE2, a subunit of RCC III, and DRP1, was significantly decreased in the neuroretina. *Drp1* and *Opa1*, protein-encoding genes responsible for mt quality control, were decreased in most of the samples from the RPE and neuroretina. **Conclusions:** The eyes of the minipig can be considered a potential RI model to study mt dysfunction in this disease. Strategies targeting mt protection may provide a promising way to delay the acute damage and onset of RI.

## 1. Introduction

Mitochondria (mt) are important organelles that provide more than 90% of the energy needed for cells to function. Mt dysfunctions associated with both primary and secondary mt diseases play an important role in a wide range of ocular pathologies, including retinopathies characterized by dysfunction of retinal ganglion cells (RGCs), photoreceptors, the retinal pigment epithelium (RPE), or combinations thereof. The retina is known to have significant metabolic activity, which is associated with a large number of mt in the above types of retinal cells, which provide a high ATP production necessary for cell function and survival. Due to the difficulty of obtaining these tissues from humans, a need for a representative animal model—e.g., the minipig, is eminent and can serve as a suitable alternative. The aim of this project was to characterize the mt parameters in retinal cells from minipigs exposed to IOP-induced retinal ischemia (RI). The present study will thus serve as a basis for the monitoring of the acute onset and rapid course of RI and to monitor the effects of potential therapies to prevent any RGC loss due to mt dysfunction. 

Previous studies have indicated a decreased mt function due to mechanical stress or chronic hypoperfusion resulting from elevated IOP [1,2]. Various RI animal models have been established already in cells and small animals (mice and rats) [3,4,5,6], which are anatomically less similar to the human eye as that in minipigs.

Mt dysfunction has also been demonstrated in tissue from glaucomatous eyes having higher than normal IOP. In this context, studies have shown mt permeability transition pore (mPTP) opening, increased release of calcium, decreased mt membrane potential (ΔΨm) in the trabecular meshwork [7], decreased quantity and quality of mt, disappearance of mt cristae in the iris [8], decreased ΔΨm, ROS generation, as well as lower antioxidant expression in the lamina cribrosa [9]. In DBA/2J mice, increased mt fission, matrix swelling, reduced cristae volume, cristae depletion, and release of OPA1 in optic neuronal principal cells (ONH) and RGC have been further shown [10] In addition, long-time exposure to increased IOP for 3 days has been shown to induce mt fission, disruption of the mt network, decreased ATP production and decreased ΔΨm [11,12]. Finally, mt dysfunction has been detected before RGC death occurs [13].

We hereby analyze the bioenergetic functions of mt in different parts of the retina (neuroretina, RPE and the underlying choroid) upon high IOP-induced RI in minipigs, which model highly resembles human disease. We hereby optimize the methodology of preparation and collection of material for mt examination under physiological conditions and create a set of controls for the studied parameters. This is a pilot study of the impact of acute onset increase in IOP-induced RI in minipigs, and its effect on the mt energy metabolism.

## 2. Materials and Methods

### 2.1. Animal Model and Sample Collection

The experiments complied with the ARVO statement for use of animals in ophthalmic and visual research and were performed according to the Animal Protection Law of the Czech Republic and approved by the Ethics Committee of the Czech Academy of Sciences, Prague, Czech Republic, approval IAPG CAS CZ /790/2019, 20 June2019. 

The model of RI was developed at the PIGMOD Center, Liběchov, Czech Republic. Three male Liběchov minipigs of age 15–55 months (animal 1 at the age of 55 months, animal 2 at the age of 24 months and animal 3 at the age of 15 months) were used in this pilot experiment. Animals were operated under general anesthesia by intramuscular injection of a TKX mixture consisting of Tiletamine (2 mg/kg, Zoletil 100, Virbac, Carros, France), Zolazepam (2 mg/kg, Zoletil 100, Virbac, Carros, France), Ketamine (2 mg/kg, Narketan 10, Vetoquinol, Prague, Czech Republic)), and Xylazine (0.4 mg/kg, Xylapan, Vetoquinol, Prague, Czech Republic). During the procedure, the eyes were moistened using a sterile saline solution. Hyaluronic acid (1.6% HA, FCI Protect, Paris, France) was introduced into the right eye’s anterior chamber using a Hamilton syringe (Hamilton AG, Bonaduz, Switzerland). The left eye of each pig served as a control. During the experiment, the IOP was measured accordingly using a hand-held IOP-measuring device iCARE tonometer (Icare Finland Oy, Vantaa, Finland). Minipig euthanasia (started by intramuscular injection of TKX mixture, followed by intravenous bolus application of 1% Propofol (20 mL/animal, Fresenius, Fresenius Kabi Deutschland GmbH, Bad Homburg, Germany), finished by exsanguination), enucleation of the eyes, and processing of the samples took place 72 h after the IOP elevation procedure. Immediately upon enucleation, the eye bulbs were dissected in an equatorial area with a sharp blade and the anterior eye segment and vitreous body were removed. Consequently, neuroretina, RPE and choroid layers were gently separated. Transmission electron microscopy (TEM) samples were immediately upon enucleation fixed in 2.5% Paraformaldehyde in buffered PBS (PBS, BioWhittaker, Lonza, Walkersville, MD, USA). For biochemical analysis, the neuroretina was mechanically separated and the RPEs were released from the Bruch’s membrane by using 0.25% trypsin (Merck KGaA, Darmstadt, Germany) applied for 20 min, and then washed in a PBS solution. Consequently, all samples for functional and protein analyses were immediately frozen in liquid nitrogen after collection and stored at −80 °C until further processing. Control samples of RPE, neuroretina, choroid, frontal cortex and basal ganglia for the RCC activity reference range were collected according to the same protocol described above.

### 2.2. Ocular Parameters 

**Figure 1:**
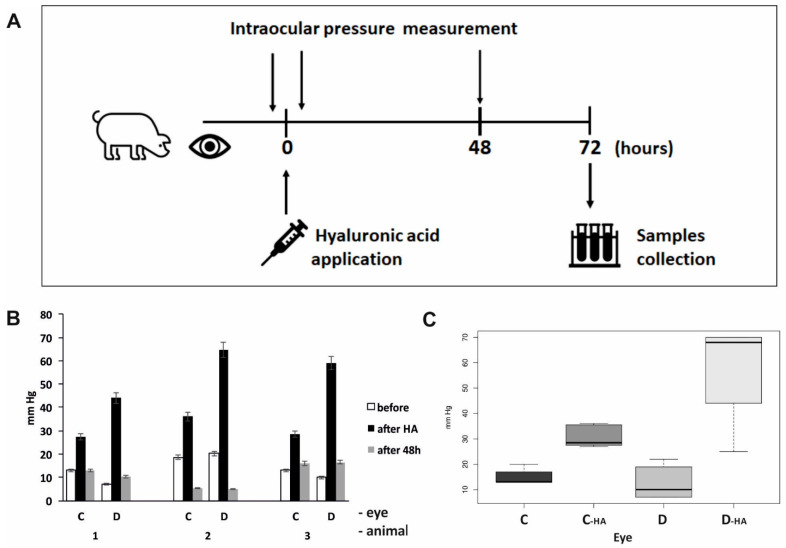
Monitoring of intraocular pressure during an experiment by iCare tonometer. (**A**) A simplified time chart of the experimental arrangement; (**B**) Intraocular pressure values in individual eyes. Means of minimum of six values are shown. HA—hyaluronic acid, C—control left eye, D—right eye after acute intraocular pressure elevation. (C) Differences between the pressure changes were analyzed using a linear mixed-effect model. Analysis was performed in a statistical package R, version 4.2.1. Before hyaluronic acid application, no difference between the control (C, *n* = 14) and treated eyes (D, *n* = 17) could be observed (*p* = 0.6206). Increase in the intraocular pressure during the experiment was significantly higher in treated right eyes (D-HA, *n* = 18) compared to control left eyes (C-HA, *n* = 12) (*p* = 0.0000).

IOP was recorded using an iCARE IC100 tonometer (Icare Finland Oy, Vantaa, Finland) before and after the application of HA in the anterior chamber of the right eye in the minipigs, as well as after 48 h (Figure 1). Spectral-domain optical coherence tomography (OCT) scanning was performed using iVue (Optovue, Fremont, CA, USA) in order to examine the condition of the retina after the treatment procedure and at 48 h. For fundus imaging under the same conditions, a non-mydriatic fundus color camera—iCam (Optovue, Fremont, CA, USA) was used. 

### 2.3. Ultrastructural Analyses

Following fixation, the retinal tissue was dehydrated using ethanol (sequential series). Then, the samples were incubated in propylene oxide and cut in Durcupan Epon (Durcupan Epon: propylene oxide 1: 1 for 2 h, Durcupan Epon: propylene oxide 1: 3 overnight). The polymerized blocks were divided on an Ultracut Reichert microtome. Briefly, 600–900A thick slices were stained using lead citrate and uranyl acetate [14]. Images were acquired using a JEOL JEM 1400 (Plus) transmission electron microscope (TEM) (JEOL, Peabody, MA, USA). At a minimum, two sections were analyzed in each cell layer of the eye, and 50 cells in each section were reviewed to evaluate mt in individual tissue layers.

### 2.4. Homogenization and Isolation of the Mitochondrial Fraction

A 10% homogenate (*w*/*v*) was prepared from frozen tissue in KTEA medium (150 mM KCl, 50 mM Tris-HCl, 2 mM EDTA, pH 7.4, and 0.2 g/mL aprotinin at 4 °C using a Potter-Elvehjem homogenizer (Bellco Glass, Inc., Vineland, New Jersey, USA) The post-nuclear supernatant (PNS) was recovered from the homogenate by centrifugation at 600× *g* for 10 min at 4 °C and filtered through a nylon mesh. The mt were sedimented by centrifugation of the residual PNS at 10,000× *g* for 10 min at 4 °C. The pellets were washed with KTEA medium, centrifuged again in the same conditions, and finally resuspended in KTEA at a protein concentration of approximately 20 mg/mL. The aliquots of the homogenate or mt were stored at −80 °C and used for subsequent analyses.

### 2.5. Functional Characterization of Mitochondria

Activities of NADH-coenzyme Q10 oxidoreductase (NQR, complex I), succinate-coenzyme Q10 oxidoreductase (SQR, complex II), coenzyme Q10—cytochrome c oxidoreductase (QCCR, complex III), cytochrome c oxidase (COX, complex IV), and citrate synthase (CS) were determined spectrophotometrically [15]. The total content of coenzyme Q10 was determined by HPLC [16].

### 2.6. Protein Analysis

Selected subunits of oxidative phosphorylation system (OXPHOS) complexes and proteins interacting in mt dynamics were analyzed by the electrophoretic method (Glycine-SDS-PAGE), based on glycine-Tris buffer systems and Western blotting using monoclonal antibodies (Abcam, Cambridge, Cambridgeshire, United Kingdom; BD Transduction Watertown, New York, USA; Cell Signaling, Danvers, MA, USA). Separations were performed on 12% glycine gels. Signal quantification was obtained using a Syngene imaging system (Syngene, Cambridge, UK) and evaluated using Quantity one 1D-Analysis Software (Biorad, Hercules, California, USA) [17,18].

The following antibodies were used: Respiratory chain: Complex I subunit NDUFA9: anti-NDUFA9 antibody (ab14713, Abcam; 1:2000); Complex I subunit NDUFB6: anti-NDUFB6 antibody (Ab110244, Abcam; 1:2000); Complex II subunit SDHa monoclonal antibody (MS204, Mitosciences, Eugene, OR, USA; 1:4000); Complex II subunit SDHb monoclonal antibody (Ab14714, Abcam; 1:2000); Complex III anti-ubiquinol-cytochrome *c* reductase core protein II antibody (MS304, Mitosciences 1:5000); Complex IV anti-COX IV antibody (Ab14744, Abcam; 1:1000); Complex V anti-ATP50 antibody (MS507, Mitosciences; 1:2000).

Mt quality control proteins: purified mouse anti-OPA1 antibody (612606, BD Transduction Laboratories; 1:1000); anti-DRP1 antibody (ab56788, Abcam; 1:500); anti-MFN1 antibody (ab57602, Abcam 1:1000); Protein β-actin (4970S, Cell signaling, Danvers, MA, USA, 1:1000) was used as a loading control.

### 2.7. mRNA Expression Studies

RNA was isolated from tissues using the TRI Reagent^®^ solution (Molecular Research center, Cincinnati, OH, USA)—a complete ready-to-use reagent for the isolation of total RNA. Isolation was processed according to the manufacturer’s protocol. For analyzing the quantity and quality of total RNA, NanoDrop 2000 (ThermoFischer, Waltham, Massachussets, USA) was used. RNA after isolation was solubilized in RNA-free water. Samples with RNA were stored at −80 °C. Transcription to cDNA was performed using LunaScript^TM^ RT SuperMix Kit (New England Biolabs, Ipswitch, MA, USA). cDNA was stored at −20 °C.

### 2.8. RT-PCR Studies

Quantification of the expression of selected genes using TaqMan assays with TaqMan probes FAM-MGB (DNML1 Ss06877558_m1, Sqstm1 Ss06902691_m1, OPA1 Ss06876119_m1, and RPS18 Ss03391030_g1 serve as a control gene) was performed by qPCR (CFX96 Touch Real-Time PCR Detection System) [15]. Each sample used for qPCR contained a cDNA template, TaqMan Gene Expression Master Mix, selected probe, and water to the final volume of up to 20 µL. Thermal-cycling protocol was performed according to the manufacturer’s instructions. Gene expression was quantified by the ∆ Ct method using a reference gene. For each gene, the samples were analyzed in triplicates.

### 2.9. Statistics

Differences between the pressure changes were analyzed using a linear mixed-effect model. Differences in further observed variables between the control left eye and right eye after acute intraocular pressure elevation were evaluated using a paired t-test. *p*-values less than 5% were considered statistically significant. Analysis was performed in a statistical package R, version 4.2.1. and GraphPad Prism 9.3.

## 3. Results

### 3.1. Ocular Examinations

The measured values of the IOP showed an increased IOP after the application of HA into the anterior chamber of the right eye in comparison to the control left eye (*p* < 0.0001). Consequently, the IOP values were normalized to the levels recorded before HA application in both eyes after 48 h post-treatment (Figure 1). 

Using both noninvasive examination methods such as OCT scanning and fundus imaging, no changes in the retina of treated eyes could be observed in comparison to the untreated ones (Supplementary Figure S1). 

### 3.2. Ultrastructural Analyses

**Figure 2:**
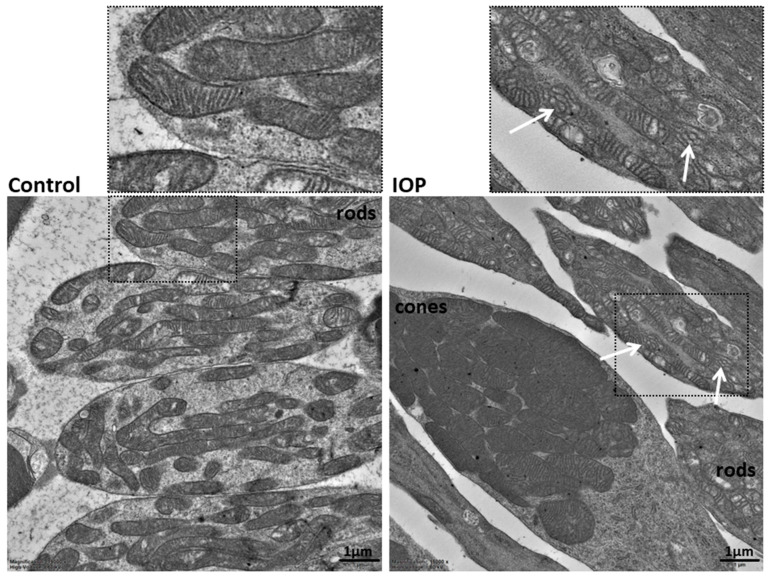
Ultrastructural features of the mitochondria in the rods of the retinal ischemia-induced (**right** image) and control (**left** image) eye. The cristae of the mitochondria are compact and narrow in the control eye. White arrows show the presence of dilated cristae with abnormal shapes in the mitochondria of the high-pressure-induced retinal ischemia model. Two ultrathin sections of the neuroretina were observed, 64 RI cells and 56 control cells were used to evaluate the mitochondrial phenotype in rods, and the phenotype appeared homogeneous throughout the sample. TEM, 15,000×.

TEM of the neuroretina showed ultrastructural changes in the mt of high IOP-induced RI eyes. Decreased number of cristae, dilated and swollen cristae with abnormal shapes were detected in the RI group (Figure 2). In total, 64 RI cells and 56 control cells were used to evaluate mt in rods, and the phenotype appeared homogeneous throughout the sample, while the mt of RI cones compared to control groups differed: they contained a lower density of cristae and appeared more swollen compared to the controls. Similar mt changes were also observed in the deeper layers of the retina.

### 3.3. Functional Analyses of Mitochondria

The control minipig neuroretina revealed measurable RCC activities that were comparable to that of identical enzymes in isolated brain homogenates and/or mt of minipigs (Supplementary Figure S2). 

**Figure 3:**
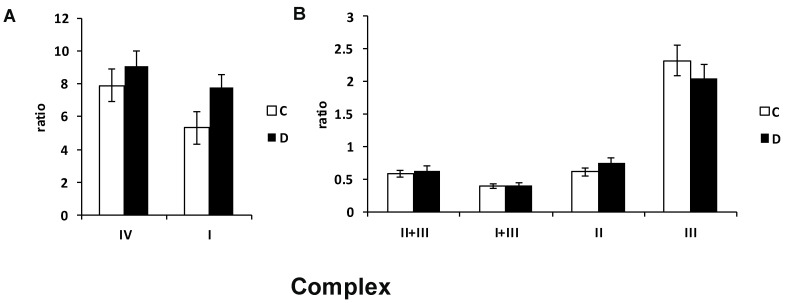
Activities of the respiratory chain complexes in the retinal pigment epithelial cells (RPE) of the minipig eyes after acute high intraocular pressure-induced retinal ischemia. Increased complex I and mildly increased complex IV (**A**) and mildly decreased complex III activity (**B**) were detected in diseased RPE cells in comparison to controls. Activities of the respiratory chain complexes were measured spectrophotometrically in homogenate, normalized values to citrate synthase are shown. I—respiratory chain complex I, NADH: ubiquinone oxidoreductase; II—complex II, succinate: coenzyme Q reductase; III—complex III, ubiquinol: cytochrome c oxidoreductase; IV—complex IV, cytochrome c oxidase; I + III, complex I + III, NADH: cytochrome c reductase; II + III, complex II + III, succinate: cytochrome c reductase. C—control eye, D—eye after acute intraocular pressure elevation. *n* = 3. Each sample was analyzed in doublets.

Analyses of the specific activities of individual RCCs in the RI group revealed increased complex I and slightly increased complex II and IV activity, whereas complex III showed a decreased tendency of activity in the RPEs of the RI-induced group (Figure 3). No differences between the left and right eyes were detected for RCC activities in the neuroretina and choroid layers.

**Figure 4:**
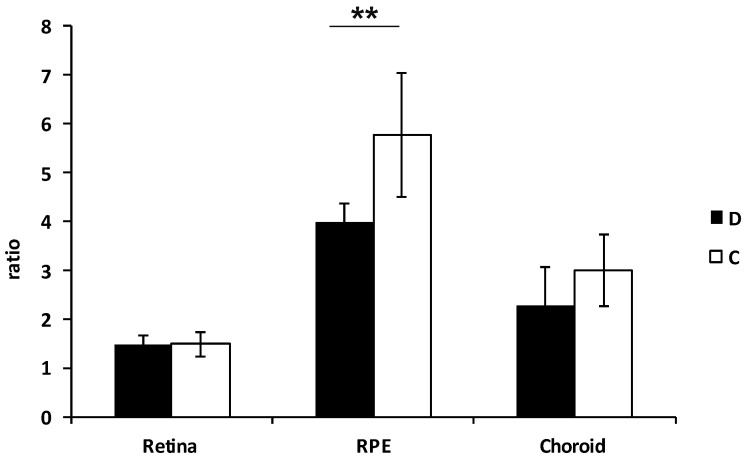
Total coenzyme Q10 content in the different layers of the minipig high intraocular pressure-induced retinal ischemia eyes. The total Q10 content in the tissue homogenate was determined using HPLC with UV detection at 275 nm. Values of Q10 content normalized to citrate synthase are shown. D—diseased eyes, C—controls. *n* = 3. Each sample was analyzed in doublets.** *p* < 0.001.

Furthermore, the total content of coenzyme Q10 was markedly decreased in the RPE (*p* < 0.001) and choroid of the RI group (Figure 4).

### 3.4. Expression Studies

**Figure 5:**
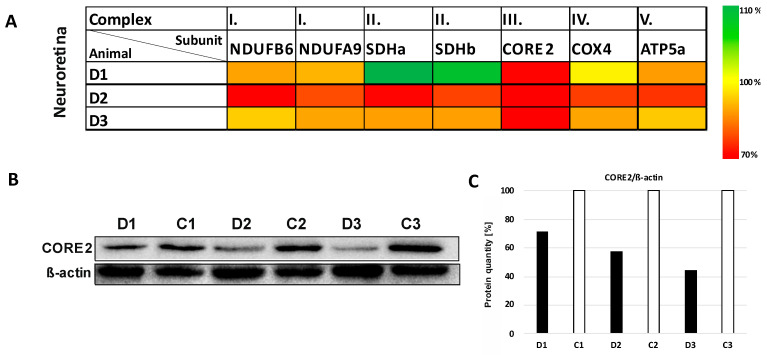
Changes in the protein expression of selected subunits of oxidative phosphorylation system complexes in the neuroretina layer of minipig eyes after high intraocular pressure-induced retinal ischemia. (**A**) The heatmap shows significant reduction in CORE2 protein (structural subunit of complex III). The results of SDS/WB evaluation were normalized to β-actin and compared to the left eyes of each minipig. D1, D2 and D3 are eyes after high intraocular pressure-induced retinal ischemia. The scale on the right shows the degree of the protein level change. (**B**) Decreased level of CORE2 was detected in neuroretina compared to the control samples (*p* < 0.0001). Lysates from homogenates of diseased/retinal ischemia (D1, D2, D3) and control (C1, C2, C3) eyes were analyzed by SDS-PAGE/WB. Briefly, 10 µg of total protein was separated in 12% glycine gel and detected. Representative SDS/WB is shown. (**C**) Quantification of the Western blot signal (shown in panel (**B**). The intensity of the bands was quantified by Quantity One software and their values were normalized to β-actin. Further comparisons of groups (control eyes versus eyes after intraocular pressure-induced retinal ischemia) are shown in Supplementary Figure S3.

To determine the actual steady-state level of RCC complexes, the selected subunits for each RCC were analyzed using SDS/WB in the neuroretina. In general, all analyzed subunits (except for complex II) in the RI model were found to be slightly to significantly decreased (NDUFB6 and COXIV, *p* < 0.01; NDUFA, *p* < 0.001). The most profound deficiency was detected for the subunit of complex III—CORE2 up, to 45% of control (*p* < 0.0001) (Figure 5 and Supplementary Figure S3).

**Figure 6:**
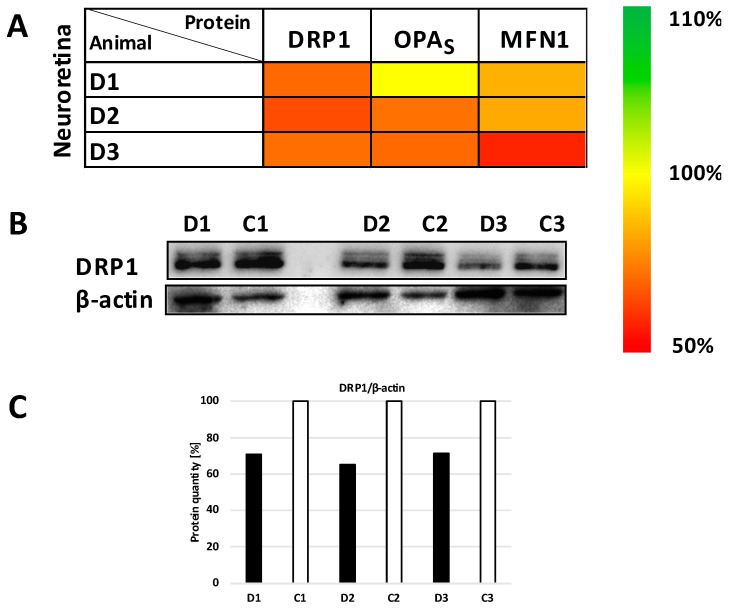
Changes in mitochondrial quality control proteins content in neuroretina layer of minipig eyes after high intraocular pressure-induced retinal ischemia. (**A**) The heatmap of Dynamin-related protein 1 (DRP1), short form of optic atrophy (S-OPA) and Mitofusin 1 (MNF1) expression. The results from SDS/WB evaluation were normalized to β-actin and compared to the control eye of each minipig. Scale on the right shows the degree of the expression change. D1, D2 and D3 are eyes after high intraocular pressure-induced retinal ischemia. (**B**) A decreasing trend of DRP1 protein content after normalization to β-actin was found in neuroretina after intraocular pressure elevation compared to control samples (*p* < 0.01). Lysates from homogenates of diseased/retinal ischemia (D1, D2, D3) and control (C1, C2, C3) eyes were analyzed by SDS-PAGE. Briefly, 10 µg of total protein was separated in 12% glycine gel and detected. Representative SDS/WB is shown. (**C**) Quantification of the Western blot signal (shown in panel (**B**)). The intensity of the bands was quantified by Quantity One software and their values were normalized to β-actin. D1, D2 and D3 are right eyes of minipig 1–3 after high intraocular pressure-induced retinal ischemia; C1, C2 and C3 are left control eyes of each minipig. Further comparisons of groups (control eyes versus eyes after intraocular pressure-induced retinal ischemia) are shown in Supplementary Figure S4.

**Figure 7:**
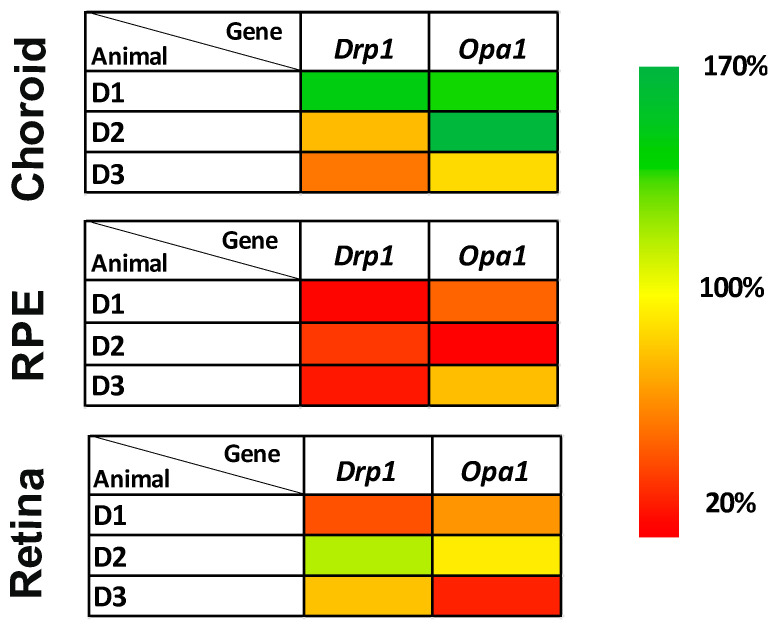
Expression of selected mRNA in different layers of minipig eyes after high intraocular pressure-induced retinal ischemia. The heatmaps show different gene expressions in each layer of the eye. Diseased/retinal ischemic right eyes were compared to control left eyes and normalized to reference gene *Rps18*. The changes in selected genes are most profound in the RPE layer. *Drp1 (Dnm1L)* gene is decreased in RPE (*p* < 0.0005), signifying potential DRP1 inhibition or disruption of the balance of mitochondrial dynamics. D1, D2 and D3 are diseased/retinal ischemia right eye of each minipig after intraocular pressure elevation. Scale on the right shows the degree of the expression change. Further comparisons of groups (control eyes versus eyes after intraocular pressure-induced retinal ischemia) are shown in Supplementary Figure S5.

The levels of representative proteins for mt dynamics were also tested. OPA1 (total and short isoforms), MFN1 and DRP1 decreased in the RI neuroretina to 60–80% (*p* < 0.01) (Figure 6 and Supplementary Figure S4). In parallel, significantly reduced expression to 25–50% of *Drp1* and *Opa1* genes was confirmed in the RPEs of the RI eyes (*p* < 0.001 and *p* < 0.01, respectively) (Figure 7 and Supplementary Figure S5). *Opa1* gene was significantly decreased in the neuroretina (*p* < 0.05).

## 4. Discussion

The retina is the most metabolically active tissue in the body, with the highest consumption of energy per unit area of tissue, and in a similar range to the brain [19]. More than 90% of energy in the cells is produced by the oxidative phosphorylation process within mt. Control minipig neuroretina revealed well-measurable activities of the RCCs, and those values were comparable to the activities of the identical enzymes isolated from brain homogenate and/or mt of minipigs (Supplementary Figure S2).

Impairment of the mt structure and function may be strongly linked to the pathogenesis of RI. The current model is based on the assumption that increased IOP and RI would work in a similar manner as middle cerebral artery occlusion, but only in the eye, without causing reduced blood flow in the brain. Such damage has been accompanied by mt dysfunction and RGC death; oxygen-glucose deprivation similarly can induce mt dysfunction and cell death in RPEs [20]. 

In the RI model, disturbances of RCC function were found. Increased complex I activity could be detected. A limited amount of substrates during ischemia could shift the soft equilibrium of the active/deactive form of complex I and maintain the enzyme partially in an active state or activation of the enzyme upon tissue reoxygenation may act as an intrinsic protective mechanism [21]. Mild decreased complex III activity and significantly lowered content of CORE2 subunit of complex III were found in diseased RPE in comparison to controls. Complex III transports electrons from coenzyme Q to cytochrome *c* within RC and consists of 11 subunits with an active center containing hemes. Chen et al. have shown that ischemia/reperfusion (I/R) injury in mice cardiomyocytes can cause significant heme components integrity damage and a decrease in complex III activity [22]. Previous studies have also identified I/R-mediated enhanced sulfonation of the specific cysteine residues as a likely antioxidant defense mechanism against oxidative stress arising from I/R. Increased sulfonation following pathological conditions after I/R has been detected also on CORE2 containing a unique thiol at C191 [22]. We can suggest that some analogical mechanisms may have had an impact on complex III in our RI model. Moreover, it should be noted that the CORE2 subunit is important for the stability of complex III.

Next, we analyzed CoQ10—a lipid-soluble component of the inner mt membrane that plays a key role in electron transport within RC. The CoQ10 creates a proton (H^+^) gradient across the inner mt membrane by transporting H^+^ from the mt matrix to the intermembrane space. Furthermore, CoQ10 also serves as a powerful antioxidant in its reduced form (CoQ10H_2_), which in turn protects the cell from oxidative stress [23]. Finally, CoQ10 acts as an H^+^ carrier to enable lysosomes to carry out their function in clearing cellular debris and maintaining intracellular integrity [24]. Total coenzyme Q10 content in the RPEs in the RI model was profoundly decreased. In a previously established increased IOP RI model, a decreased ΔΨm was shown [11]. It was thereby proven that the decreased ΔΨm and mt ATP levels caused by uncoupler FCCP treatment or MERRF (myoclonic epilepsy with ragged red fibers) mutation could suppress the COQ5 protein maturation [25]. COQ5 is a human enzyme required for CoQ biosynthesis and its impairment disrupts the formation of COQ protein complex (Q-synthome) near the inner mt membrane and thus causes decreased biosynthesis of CoQ10 [25,26]. An analogous mechanism could be the cause of reduced coenzyme Q10 levels in our model.

Patients with primary CoQ10 deficiency may have retinopathy as part of their syndrome, which suggests that CoQ10 may play an important role in the pathogenesis of retinal conditions [27,28]. Furthermore, improved bioenergetic status of RGCs following high-dose of CoQ10 supplementation (1.2–2.4 g/day) has been found in age-related macular degeneration and other age-related degenerative disorders, further highlighting its importance in retinal tissue [29,30].

The mt quality control process, fusion and fission, should be in soft balance in physiological conditions. In the RI model, the DRP1 protein—the main player in mt fission, was found to be decreased together with a decreased gene expression. In studies where long-time sustained elevated IOP has been induced, an increased DRP1 has been found [31]. We suggest a possible opposite finding in our RI model—depressed fission may be a biomarker for defense cell mechanisms in the early stages of RI development, to maintain enough substrates and energy production and promote the survival of the neuroretina cells. Similar results have been shown by inhibition of DRP1 activity and reduction in oxidative stress [31].

In the neuroretina and RPEs, decreased OPA1 and MNF1 levels were detected—two proteins that are the main players in the fusion of mt. It is still not exactly known how the long isoforms of OPA1 participate in the prolongation of the mt, while short isoforms are responsible for the maintenance of the inner cristae structure [32,33]. Decreased content of OPA1 short isoforms is in concordance with the ultrastructural analysis, where the presence of dilated cristae with abnormal shape was detected in the mt of the RI model. 

We present a simple model for high IOP-induced RI in a large animal model (minipigs) in its early stages of development. The advantages of the minipig model are the similar anatomy and size of the pig eyes to those in humans, as well as the similar IOP baseline and increased values; the circulation and blood supply to the retina in minipigs also resembles that in humans. A disadvantage would be the not so verse availability of minipig facilities worldwide and the higher costs associated with doing animal experiments.

The advantages of the minipig eye have already been described in one of our recent publications [34], as well as the minipig model of Usher disease [35]. In addition, the minipig model has proven to be excellent in other neurodegenerative disorders, such as Huntington’s disease, where experimental treatment is already being tested [36].

## 5. Conclusions

We show that the RPEs are the most vulnerable or impaired tissue during RI. The function of the oxidative phosphorylation system complexes was imbalanced, and oxidative stress presence was detected.

The finding of declining levels of DRP1 protein in the neuroretina as well as a decline in the *Drp1* gene in RPEs suggest inhibition of the DRP1 expression in these layers of the eye. DRP1 inhibition can promote the survival of cells in the neuroretina. Impaired ultrastructure of the mt together with decreased OPA1 protein levels in the neuroretina, as well as decreased expression of *Opa1* gene in the neuroretina and RPE, together with DRP1 changes, indicated that these molecules can possibly serve as biomarkers of subtle changes in the early stages of RI development. The low content of CoQ10 in the RPEs may contribute to the supposed increase in oxidative stress and the decreased defense ability of the cells. Further studies are necessary to examine in detail the processes occurring during the early stages of RI development since various strategies targeting mt protection might provide a promising way to delay the onset of I/R damage due to RI or protect the retinal cells against such damage.

## Data Availability

All data of this study will be made available upon request to the corresponding author.

## Supplementary Materials

The following supplementary information can be downloaded at: https://www.mdpi.com/article/10.3390/biom12101532/s1, Figure S1. Fundus imaging and OCT scans of the retina before treatment, immediately after treatment, and 48 h post-treatment of the pig right eye; Figure S2. Activities of respiratory chain complexes and citrate synthase in various tissues of control pig’s eyes in comparison to brain tissue; Figure S3. Differences of respiratory chain protein expression between control left eye and right eye after acute intraocular pressure elevation; Figure S4. Differences of mitochondrial quality control protein expression between control left eye and right eye after acute intraocular pressure elevation; Figure S5. Differences of genes expression between control left eye and right eye after acute intraocular pressure elevation.
