# Supplementary figures and images for: Mitochondrial Dysfunction in a High Intraocular Pressure-Induced Retinal Ischemia Minipig Model
Source: Biomolecules. 2022 Oct 21;12(10):1532. doi: 10.3390/biom12101532 (PMC9599919; doi:10.3390/biom12101532)

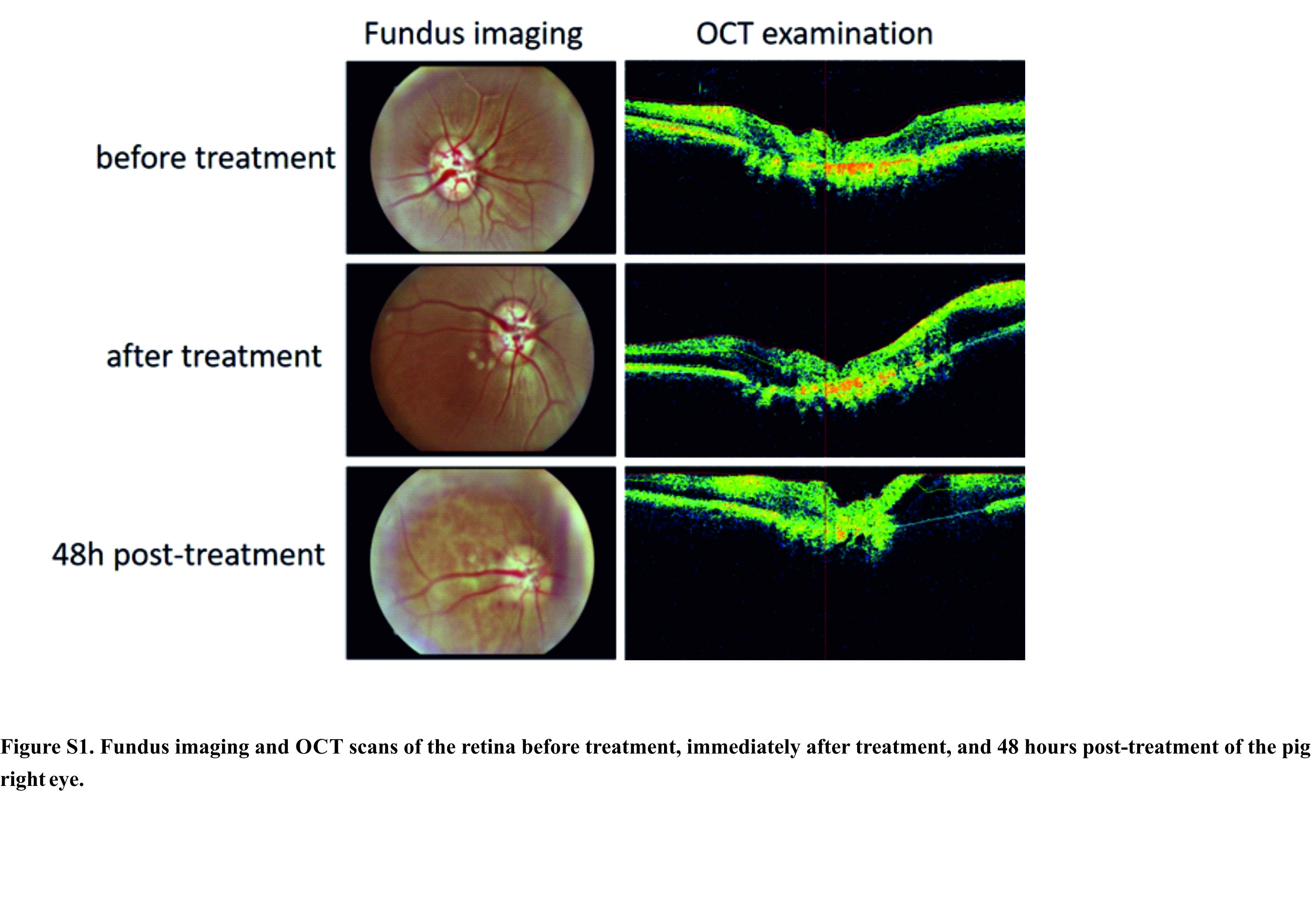

Supplement: Supplementary file 1 [file biomolecules-12-01532-s001.zip › Supplementary Figure S1.jpg]

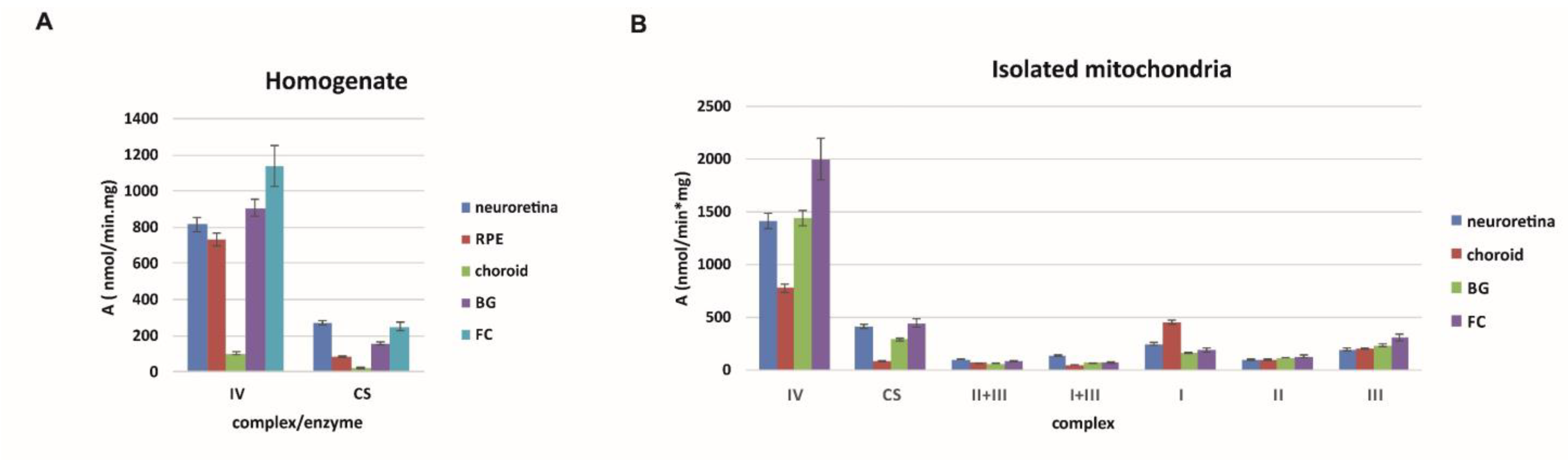

Supplement: Supplementary file 1 [file biomolecules-12-01532-s001.zip › Supplementary Figure S2.tif]

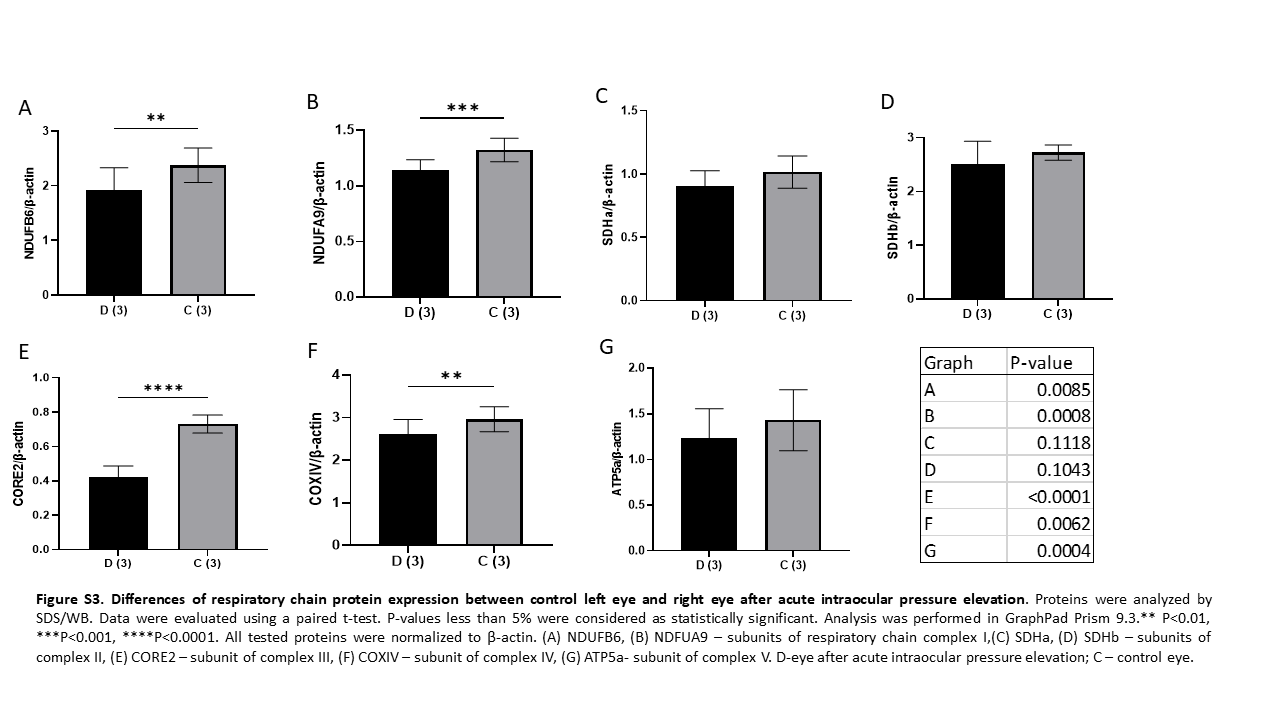

Supplement: Supplementary file 1 [file biomolecules-12-01532-s001.zip › Supplementary Figure S3.tif]

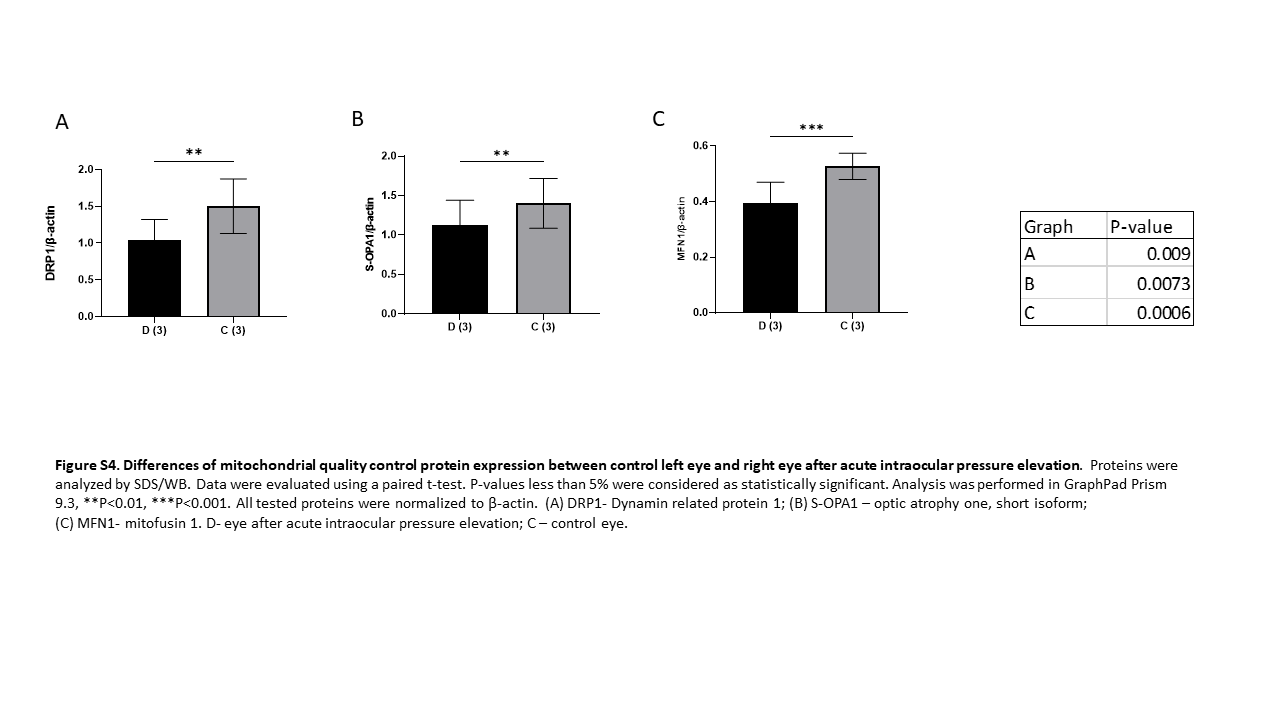

Supplement: Supplementary file 1 [file biomolecules-12-01532-s001.zip › Supplementary Figure S4.tif]

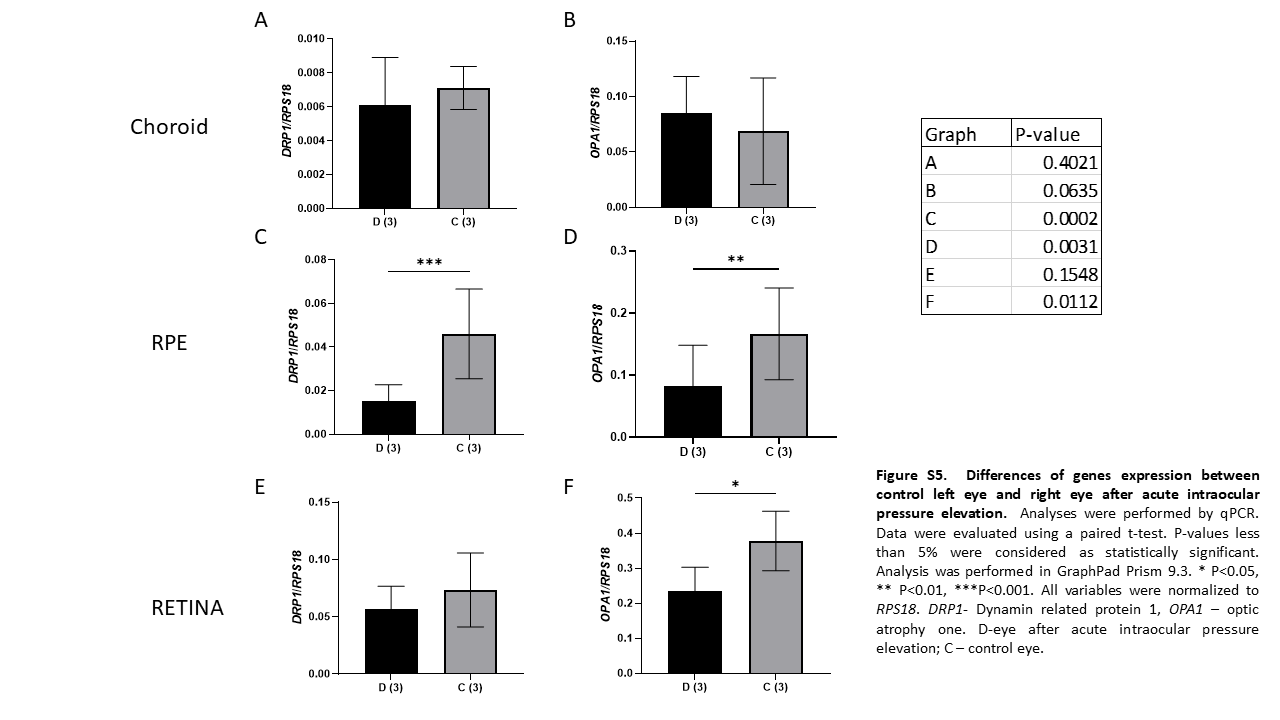

Supplement: Supplementary file 1 [file biomolecules-12-01532-s001.zip › Supplementary Figure S5.tif]
